# Positive impacts of *Nannochloropsis oculata* supplementation on gene expression of immune and antioxidant markers and metabolic profile of Barki sheep in the transition period and lipogenic effects on progeny

**DOI:** 10.1007/s11259-024-10392-2

**Published:** 2024-05-04

**Authors:** Ahmed El-Sayed, Eman Ebissy, Ahmed Ateya

**Affiliations:** 1 Department of Animal Health and Poultry, Animal and Poultry Production Division, Desert Research Center (DRC), Cairo, Egypt; 2 Department of Development of Animal Wealth, Faculty of Veterinary Medicine, Mansoura University, Mansoura, Egypt

**Keywords:** Peripartum, *Nannochloropsis oculata*, Antioxidants, Immunity

## Abstract

*Nannochloropsis* species should be given priority when it comes to microalgae that should be added to feed since they are suitable for intense culture and have a high concentration of PUFAs (especially EPA), antioxidants, and certain vitamins. This study investigated the possible immune and antioxidant impacts of *Nannochloropsis* supplementation on Barki ewes during transition period and their newly born lambs. Three weeks prior to the expected time of lambing, the researched ewes were divided into two equal groups of thirty ewes each. The second group, on the other hand, was fed the same base diet as the first group plus 10 g of commercially available *Nannochloropsis* powder per kg of concentrate, given daily to each ewe’s concentrate. Findings revealed that supplementation of ewes with *Nannochloropsis* significantly up-regulated the expression pattern of immune (*NFKB, RANTES, HMGB1, TNF-α, IRF4, TLR7, CLA-DRB3.2, IL1B, IL6, CXCL8, S-LZ*, and *Cathelicidin*), and antioxidant (*SOD1, CAT, GPX1, GST, ATOX1, Nrf2* and *AhpC/TSA*) markers in ewes post-lambing and their newly born lambs. Additionally, mRNA levels of lipogenic (*ACACA, FASN SCD, LPL*, and *BTN1A*) markers were significantly up-regulated in lambs from supplemented ewes than control ones. There was a significant increase in the WBCs, Hb, RBc count, serum level of glucose, total protein, triacylglycerol and total cholesterol, GPx, catalase, IL1α and IL6 with significantly decreased serum level of TNF-α and MDA in supplemented ewes after lambing as compared with control ones. There was also a significant increase in WBCs, Hb, RBc count, birth weight and body temperature with significantly decreased in the serum levels of TNF-α and stillbirth of newly born lambs from supplemented ewes as compared to other lambs from control ones.

## Introduction

Ruminant researchers have identified the transition period as occurring three-weeks around parturition (Sucupira et al. 2019). Due to increased nutritional needs to combat the foetus’ growth and the first milk’s production during those time, significant metabolic and endocrine changes frequently take place (Sucupira et al. 2019). These characteristics may make the animal more susceptible to immunological suppression, the generation of reactive oxygen species (ROS), and the development of possible illnesses such nutritional, metabolic and viral diseases especially pregnancy toxemia, hypocalcaemia and hypomagnaesemia (Caroprese et al. 2006). This is particularly true in the event of inadequate food intake and/or non-metabolic adaptation to the altered physiological state. According to, (Sucupira et al. 2019) the increased creation of ROS and the demand for endogenous and exogenous antioxidant factors might cause oxidative stress, impair neutrophil activity, antibody responses, and increase cytokine secretion by immune cells.

There are studies that have determined that feeding with algae is a behavior that has existed for many years, especially in species of sheep kept near marine coastlines (EL-Sabagh et al. 2014). Tiny doses of microalgae supplementation exhibited positive effects on animal physiology, productivity, and feed conversion by improving gastrointestinal and immune processes (Camacho et al. 2019). The diverse group of autotrophic and photosynthetic microorganisms known as microalgae possesses a number of special biological traits, such as high photosynthetic energy transfer efficiency and the capacity to synthesize biologically complex substances like lipids, proteins, carbohydrates, pigments, and polymers, (De Morais et al. 2015) have a high content of bioactive compounds, including protein, polysaccharides, (Mohamed 2008) and vitamins such as vitamins A, C, E, K, thiamine (B1), pyridoxine (B6), riboflavin (B2), nicotinic acid, biotin, and tocopherol (Khan 2018) excellent adaptability to various environments and capability of producing a broad variety of bioenergy (Levering et al. 2015). The antioxidant defence system is strengthened by the presence of natural antioxidants found in microalgae, including phenols, flavonoids, carotenoids, and chlorophyll (Ben et al. 2017). In fact, when their diets were supplemented with microalgae, fattening lambs (EL-Sabagh et al. 2014) all showed an improvement in their antioxidant status.

An imbalance between the formation of reactive oxygen species (ROS) and the ability of antioxidant systems to neutralize those ROS can result in oxidative stress in ruminants due to a variety of environmental, physiological, and nutritional factors (Sies 1991). Feedstuffs with high levels of naturally occurring antioxidant compounds may protect animals from oxidative stress and alleviate consumers’ safety concerns. The body frequently uses a range of antioxidant processes, both enzymatic and non-enzymatic (metabolites, for instance), to combat oxidative stress (Ye et al. 2015). Numerous endogenous enzymes, present in both blood and milk, including glutathione reductase (GR), superoxide dismutase (SOD), catalase (CAT), glutathione transferase (GST), and glutathione peroxidase (GSHPx) (Board and Menon 2013), constitute the primary constituents of the intracellular antioxidant defense mechanisms that govern the accumulation of reactive oxygen species (ROS) in tissues (Sordillo 2013).

The genus *Nannochloropsis* has six distinct species, including *N. gaditana, N. salina, N. limnetica, N. granulata, N. oceanica, and N. oculata.* It is a species of monocellular microalga with a single chloroplast and a polysaccharide cell wall structure (DJ 2008). Numerous studies (both in-vitro and in-vivo) have demonstrated the beneficial effects of *N. oculata* on palatability, lack of toxicity, easy digestion (*N. oculata*) (Kholif et al. 2020), antioxidant actions (*Chlorella vulgaris*) (Elsheikh et al. 2018), immunity (Bule et al. 2018), anti-inflammatory and anti-cancer *(N. oculata)* (Md et al. 2018) on several animal, additionally to possibility to use as a substitute source of the conventional protein on animals diet. In addition, they serve as a reliable alternate supply of Eicosapentaenoic acid (EPA, C 20:5 n3) (Becker 2007).

Because antioxidants work together to neutralize oxidative offence, measuring the amount of each antioxidant separately does not give a good indication of the antioxidant capacity (MR 2016). Consequently, not all antioxidant defense mechanisms are necessarily affected by a lack in one antioxidant. As a result, numerous techniques have been created to determine the overall antioxidant capacity. In order to anticipate a herd’s susceptibility to production disorders, it has recently been shown that examining the gene expression of antioxidant biomarkers of the transition phase offers a trustworthy technique to monitor animal health during this crucial time (Lager and Jordan 2012).

According to Van Harten et al. 2013; to enhance genetic selection for livestock adaptation to difficult environments, differences in the expression of certain regulatory genes involved in the intermediate metabolism can be useful tools. One aspect of metabolic regulation is the transcriptional regulation of gene networks, which are collections of DNA segments that interact with nuclear receptors or transcription factors to regulate the concentration of critical enzymes in cells. The rate at which the genes in the network are translated into mRNA might be regulated by these “global” interactions. Research on the entire genome, sub-networks, or candidate genes at the mRNA level are all included in the broad field of genomics (Loor 2010).

Based on what is currently known, little is known about how *N. oculata* affected the metabolic profile and gene expression in Barki ewes during the transition phase. The objective of the current study was to ascertain the possible impacts of N. oculata supplementation on a few immune-metabolic variables and oxidative stress markers in Barki ewes during the transition phase. Clarifying the impact of *N. oculata* fed to ewes during the transition phase on the immunological and antioxidant state of their young following lambing is another goal. Our hypothesis is that variations in the investigated genes and the regulatory enzymes of the intermediate metabolism could be useful tools to enhance genetic selection for the adaptation of Barki sheep to harsh environments.

## Materials and methods

### Animals

In this study, a total of 60 multiparous pregnant Barki ewes that appeared healthy and ranged in age from 4 to 6 years (mean ± SD: 4.9 ± 0.7) and body weight from 40.5 to 64 kg (mean ± SD: 49.16 ± 6.5) were used. Based on the study station’s files and records, every ewe under investigation had three lambings before the trial started Pregnancy was confirmed using ultrasound (Samsung Medison SONOACE R3 ultrasound system, South Korea). The experiment was carried out in Alexandria, Egypt at the Mariut Research Station and Desert Research Centre. The Arab Veterinary Industrial Company (AVICO), Amman, Jordan, gave oral benendazole, a broad-spectrum anthelmintic, to each of the sheep under examination on August 1st, prior to the onset of the reproductive season in October. The dose was 10 mg/Kg BW.

The sheep in question underwent a comprehensive clinical examinationand and the body weight, body temperature of their newborn lambs were measured in accordance with the previously established protocols, (Rankins and Pugh 2012) and the results were simultaneously recorded. The percentage of dead lambs were measured. All of the animals were kept in similar housing under constant veterinary observation and were apparntly healthy, with no history of metabolic or concomitant illnesses. The ewes were kept in semi-open shaded pens and fed 750 g of concentrate feed mixture (CFM) and 750 g of alfalfa hay per head each day, with unlimited access to water. Table 1 lists the ingredients in the basic diet.

**Table 1:** Composition of the concentrate feed mixture (CFM) / 1000 kg

Ingredients	Quantity
Corn	400 kg
Wheat bran	300 kg
Soya bean	250 kg
Sodium chloride	10 kg
Calcium carbonate	20 kg
Premix	1 kg
Netro-Nill	0.5 kg
Fylax	0.5 kg

### Experimental design

Two equal groups of thirty multiparous ewes each were randomly assigned to the investigated ewes three weeks before the anticipated period of lambing. While the second group received the same basal diet as the first group, but with the addition of commercially available *Nannochloropsis* powder, which was added daily to each ewe’s concentrate at a rate of 10 g of *Nannochloropsis*/kg of concentrate (EL-Sabagh et al. 2014; Tsiplakou et al. 2018) until lambing. The first group was fed the basal diet without any feed supplement and was regarded as the control group.

The Biotechnology Microalgae Culture Unit, National Research Center (NRC), Giza, Egypt, developed and graciously contributed the microalga *Nannochloropsis oculata* (*N. oculata*) used in this work. The usual F/2 Guillard’s medium was used to maintain microalgae (RR 1962). After the growing period was up, the microalgae were collected and refrigerated at 4 oC until they could be harvested by centrifugation. The technique for microalgae N. oculata extraction was used as described by (Hassan et al. 2015). The chemical composition of microalgae *N. oculata* extract was determined by gas chromatography-mass at complex laboratories of National Research Centre, Dokki, Giza, Egypt. The identification and quantitative measurements of microalgae *N. oculata* extract constituents are presented in Table 2.

**Table 2:** The quantitative measurements of ***Nanochloropsis oculata*** constituents by GC mass

**Chemical composition(g/100** **g)of microalgae***Nannochloropsis oculata*	
Moisture	7.15
Crude protein	55.78
Fat	6.61
Ash	12.29
Quantitative constituents of minerals profile (mg/100 g) in microalgae Nannochloropsis oculata	
Fe	29.35
Zn	1.02
Sodium	1862.70
Calcium	229
Potassium	798
Magnesium	173
Quantitative constituents of Amino acids profile (mg/g) in microalgae Nannochloropsis oculata	
Methionine	69.52
Cystine	17.30
Phenylanlanine	16.24
Lysine	15.20
Isoleucine	55.95
Leucine	65.11
Aspartic acid	30.16
Glutamic acid	15.07
Histidine	13.22
Tyrosine	87.69
Threonine	39.21
Valine	50.36
Serine	11.64
Glycine	9.98
Proline	31.52
Alanine	20.24
Arginine	8.56

### Blood sampling

Jugular vein punctures were used to obtain ten milliliters of blood from each ewe three weeks before to lambing as well as from both ewes and their lambs two hours after lambing. The samples were drawn into vacutainer tubes with anticoagulant (EDTA or sodium fluoride) and without anticoagulant, respectively, in order to create whole blood or serum. Blood in plain tubes was centrifuged for 15 min at 3000 rpm after being left overnight at room temperature. The EDTA blood was utilized for CBC and real-time PCR assays. Serum biochemical analyses were performed using commercial test kits in accordance with the providers’ standard operating procedures.

### RNA extraction and reverse-transcriptase PCR

Whole blood samples from ewes and their lambs were subjected to total RNA extraction using Trizol™ reagent (Invitrogen, UK), in accordance with the manufacturer’s instructions (Direct-zolTM RNA MiniPrep, catalog No. R2050). The amount of RNA extracted quantified and qualified using a NanoDrop® (ND-5000 spectrophotometer) and its integrity was evaluated by agarose gel electrophoresis. An equivalent to 1 µg of RNA was transferred to cDNA with high capacity (SensiFastTM cDNA synthesis kit, Bioline, catalog No. Bio- 65,053).

PCR amplifications were performed in a final volume of 20 µl containing total RNA template up to 1 µg, 4 µl 5× Trans Amp buffer, 1 µl reverse transcriptase and DNase free-water up to 20 µl. Reverse-transcription was done through placing the final reaction volume in a thermal cycler with the following cycling program; at 25 °C for 10 min for primer annealing, followed by reverse transcription at 42 °C for 15 min, then inactivation at 85 °C for 5 min. The samples were held at 4 °C.

### Quantitative real time PCR

Relative quantification of mRNA level of immune (*NFKB, RANTES, HMGB1, TNF-α, IRF4, TLR7, CLA-DRB3.2, IL1B, IL6, CXCL8, S-LZ*, and *Cathelicidin*) and antioxidant (*SOD1, CAT, GPX1, GST, ATOX1, Nrf2* and *AhpC/TSA*) markers was performed in blood of both ewe and newly born lambs by real-time PCR using SYBR Green PCR Master Mix (2x SensiFastTM SYBR, Bioline, catlog No. Bio-98,002). Moreover, the expression profile of lipogenic (*ACACA, FASN SCD, LPL*, and *BTN1A*) was quantified in blood of newly born lambs of both groups. The primer sequence was designed according to the PubMed published sequence of *Ovis aries* as shown in (Table 3).

**Table 3:** Oligonucleotide primers sequence, annealing temperature and PCR product size of the studied genes

Investigated marker	Primer	Product size (bp)	Annealing Temperature (°C)	AF283892.1
*NFKB*	F5′-GCCTTTGGGGACTTCTCTCC-3′ R5′- GCAGGAACACGGTTACAGGA − 3′	109	58	EF681968.1
*RANTES*	F5′- GGACGCCTTGAACCTGAACT − 3′ R5′- GTGGAATTGTGCCCTCCCAG − 3′	116	58	XM_042254827.1
*HMGB1*	F5′ACTGGTTTCTTGATCCATTTCCCT-3′ R5′-ACACGATGAGATCACGGTCC-3′	226	60	DQ153000.1
*TNF-α*	F5′-CTGCTGCACTTCGGGGTAA-3′ R5′-RTGACGTCAGGGTCTTAACCA-3′	94	58	XM_042236739.1
*IRF4*	F5′-CGCAGAGATCCCGTATCAGT-3 R5′-TGGAGCCGGCAGTCATTTTC-3	90	60	NM_001135059.1
*TLR7*	F5′-TCCATTTCCTTGCACACCGT-3′ R5′-GGGCACATGCTGAAGAGAGT-3′	121	60	EF681968.1
*CLA-DRB3.2*	F5′-′GCCTCTGATCAGGCTTCTTCT-3′ R5′- TCACACCTGCTGTGAGACCAG − 3′	120	60	NM_001009465.2
*IL1B*	F5′- TGGGACGTTTTAGAGGTGGC-3 R5′- GTCCTCGGGGTTATTCAGCC − 3′	108	60	NM_001009392.1
*IL6*	F5- TGCAGTCCTCAAACGAGTGG-3′ R5′- CCGCAGCTACTTCATCCGAA − 3′	110	58	NM_001009401.2
*CXCL8*	F5- AGTACTGTGTGGGTCTGGTG-3′ R5′-AGGAACTCGTGAATCCTGGC- 3′	250	58	GQ888735.1
*S-LZ*	F5-GGTCTGGCTTCTCAGTCAAC- 3 R5-TCAAAGACCTTGGCTTGGACA- 3	85	60	AB973433.1
*Cathelicidin*	ACGGTGAAAGAGACCGTGTG ACACTGTTTCACCAGCCCAT	87	62	NM_001145185.2
*SOD1*	F5-TGATCATGGGTTCCACGTCC-3 R5-CACATTGCCCAGGTCTCCAA-3	139	60	GQ204786.1
*CAT*	F5′-CAGTAGGAGACAAACTCAATG-3′ R5′- ACGACTCTCTCAGGAATTCTC − 3′	121	62	JF728302.1
*GPX1*	F5′-CGAGGAGATCCTGAATTGCCTGA-3′ R5′- ACCTCGCACTTTTCGAAGAGC − 3′	95	60	AJ238319.1
*GST*	F5′-TGGCTGCAGCCGGAGTGGAGTT-3′ R5′-TGGCAACGTAGTTGAGAATGGC-3′	162	64	XM_005683194.3
*ATOX1*	F5′-GCAGCCACCACCTCCTCCTCAA-3′ R5′-GTGCTCAGAGTTGATGCAGAC-3′	122	58	XM_012132956.4
*Nrf2*	F5′- GCAGTTCACTCAGTGCCATC-3′ R5′- TACCTCTCGACTTACCCCGA-3′	249	58	XM_042243029.1
*AhpC/TSA*	F5′-TGCCTCCTCAGGTAACAACA-3′ R5′-GTTGGGGAGGGTCAACAACA-3′	240	59	NM_001009256.1
*ACACA*	f5^,^- ATGTGGCCTGGGTAGATCCT-3^,^ r5^,^-ACGTAACACAAGGCTGATGGTG-3^,^	261	60	XM_004013447.1
*FASN*	f5^,^- GGAAGGCGGGACTATATGGC-3^,^ r5^,^- CATGCTGTAGCCTACGAGGG-3^,^	278	62	NM_001009254.1
*SCD*	f5^,^- GGCGTTCCAGAATGACGTTT-3^,^ r5^,^- TGAAGCACAACAGCAGGACA-3^,^	251	58	NM_001009394.1
*LPL*	F5^,^-ACCAGACTCCAACGTCATCG-3, R5^,^-GCCGGTAATCCTGTTGACCT-3,	235	59	XM_004019064.5
*BTN1A1*	F5,-AGCCTCTGATGATGGGGAGT-3, R5,-AGTTCGCCACTGTACTTGGG-3,	193	60	NM-001034034
*GAPDH*	f5^,^- TGACCCCTTCATTGACCTTC-3^,^ r5^,^- GATCTCGCTCCTGGAAGAG-3^,^	143	62	AF283892.1

NFKB = Nuclear factor kappa B; RANTES = regulated on activation, normal T cell expressed and secreted; HMGB1 = High mobility group box 1; TNFα = Tumor necrosis factor alpha; IRF4 = Interferon Regulatory Factor 4; IL1B = Interleukin 1 beta; IL-6 = Iterleukin-6; CXCL8 = chemokine (C-X-C motif) ligand 8; S-LZ = serum lysozyme; TLR7 = Toll-like receptor 7; CLA-DRB3.2 = Caprine leukocyte antigen-DRB3.2; SOD1 = Superoxide dismutase 1; CAT = Catalase; GPX1 = Glutathione peroxidase 1; ATOX1 = antioxidant 1 copper chaperone 1; GST = Glutathione S transferase; and Nrf2 = Nuclear factor-erythroid factor 2-related factor.; AhpC/TSA alkyl hydroperoxide reductase/thiol-specifc antioxidant; ACACA = Acetyl-CoA carboxylase 1, FASN = Fatty acid synthase; SCD = Stearoyl-CoA Desaturase; LPL = lipoprotein lipase; BTN1A1 = Butyrophilin Subfamily 1 Member A1 and GAPDH = Glyceraldehyde-3-Phosphate Dehydrogenase

Primer sequences, annealing temperature and the size of each amplified PCR product are shown in Table 3. The house keeping gene *GAPDH* was used as an internal control. The reaction mixture was carried out in a total volume 20 µl consisted of 10 µl 2x SensiFast SYBR, 3 µl cDNA, 5.4 µl H2O (d.d water), 0.8 µl of each primer. The PCR cycling conditions were as follows: denaturation program 94 °C for two minutes; amplification and quantification program repeated 40 cycles of denaturation temperature 94 °C for 10 s, annealing temperature for 30 s (Table 3), and extension temperature 72 °C for 20 s. At the end of the amplification phase, a melting curve analysis was performed to confirm the specificity of the PCR product. The relative expression of the gene in each sample versus a control in comparison to *GAPDH* gene and calculated according to the 2^-ΔΔCt^ method (Pfaffl 2001).

### Biochemical parameters

The following commercial kits were used on a selective chemistry analyzer (Apple 302, USA) in accordance with the standard protocol of the suppliers to quantify each of the following: triglyceride levels (Spinreact Company, Spain); aspartate aminotransferase (AST) and alanine aminotransferase (ALT) (Spectrum Company, Egypt); total protein, albumin, glucose, cholesterol, and blood urea nitrogen (BUN) (Gamma Trade Company, Egypt); and Globulin was calculated by subtracting albumin values from total serum protein. Biodiagnostic Egypt has the following CAT numbers: GP 2524 for glutathione peroxidase (GPx), GP 2529 for malondialdehyde (MDA), CA252417 for catalase (CAT), and SD 25 20 for super oxide dismutase (SOD); IL 1 alpha ELISA Kit (Ray Biotech, Inc, CAT No: ELR-IL1a), IL 6 (BOSTER BIOLOGICAL TECHNOLOGY, CAT No: EK0412) and TNF-α ELISA Kit (AVIVA SYSTEM BIOLOGY).

### Statistical analysis

H_O_: Dietary supplementation of *Nannochloropsis* could not modulate gene expression and metabolic profile of immune and antioxidant markers in ewes and their newly born lambs.

H_A_: Dietary supplementation of *Nannochloropsis* could modulate gene expression and metabolic profile of immune and antioxidant markers in in ewes and their newly born lambs.

The collected data were all represented as mean ± SEM (standard error), and SPSS version 17 was used for the statistical analysis (SPSS 2004). To compare different variables, use one-way analysis of variance and then Duncan’s multiple range tests. Values of *P* < 0.05 were deemed significant for all analyses. A chi-square test was used to test stillbirth, and the results are shown as a percentage.

## Results

### Clinical examination

Throughout the study period, the Barki ewes under investigation did not exhibit any discernible changes in their clinical condition. Every ewe under investigation had vital signs that were within the usual reference range, and they all labored and gave birth normally and without any evident clinical disease.

### Gene expression pattern of immune and antioxidant markers

**Fig. 1:**
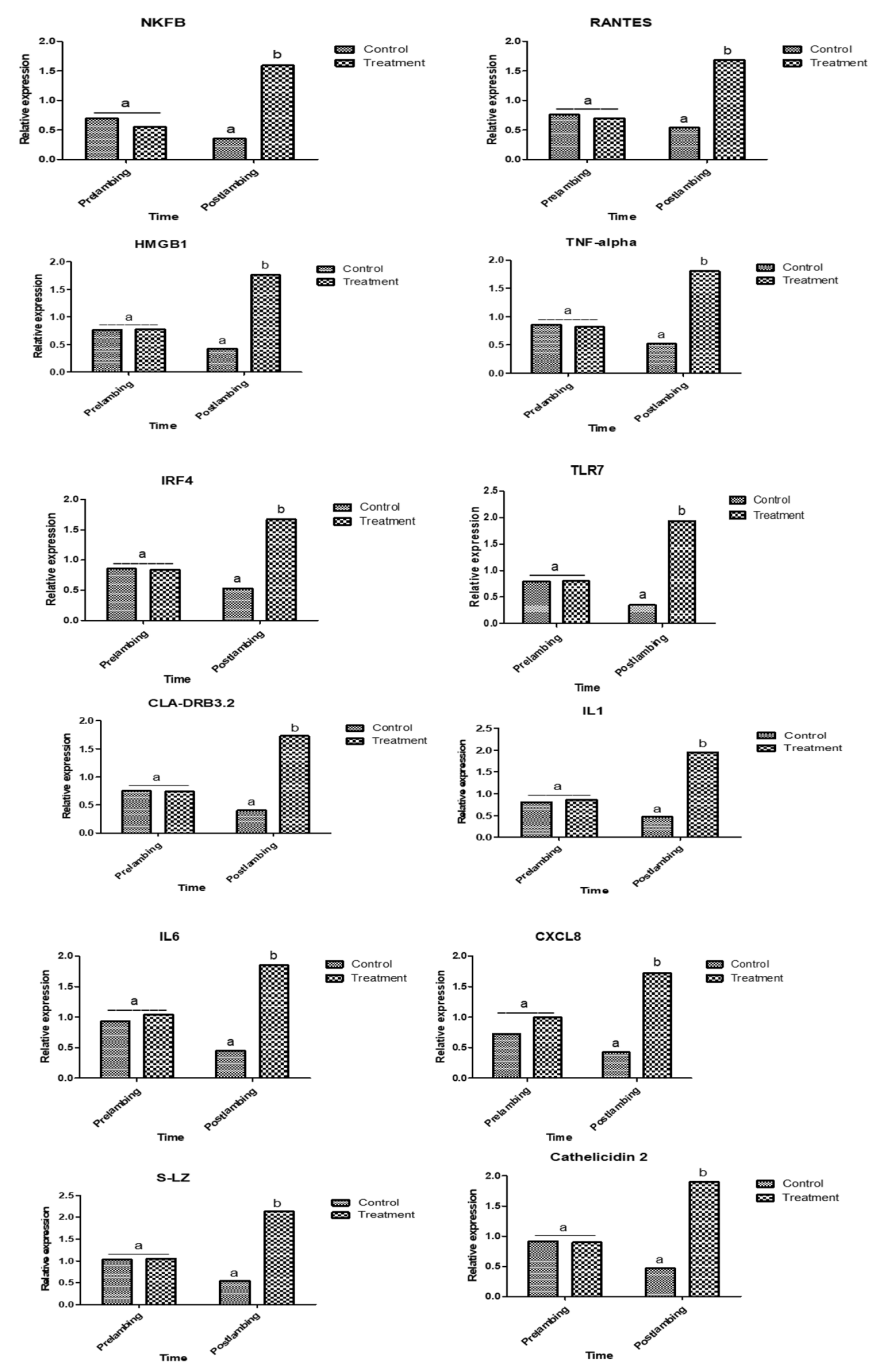
Relative expression patterns of immunity genes in Barki ewes supplemented with Nannochloropsis algae before and after lambing. Results are expressed as means ± SEM. Small alphabetical letters show significance when (*P* < 0.05)

**Fig. 2:**
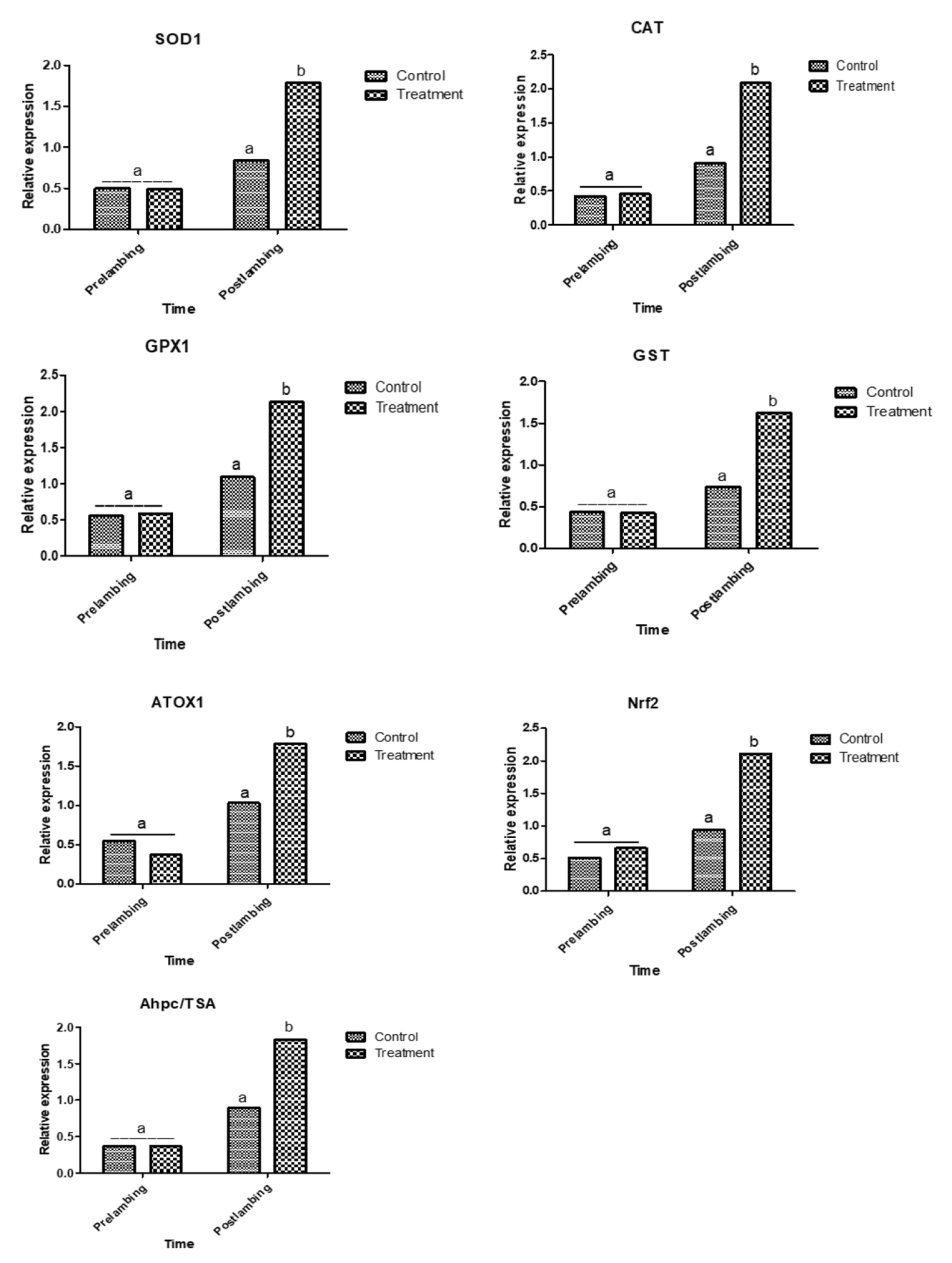
Relative expression patterns of antioxidant genes in Barki ewes supplemented with Nannochloropsis algae before and after lambing. Results are expressed as means ± SEM. Small alphabetical letters show significance when (*P* < 0.05)

Supplementation of ewes with *Nannochloropsis* could modulate gene expression profile of immune and antioxidant markers (Figs. 1 and 2). Levels of immune (*NFKB, RANTES, HMGB1, TNF-α, IRF4, TLR7, CLA-DRB3.2, IL1B, IL6, CXCL8, S-LZ*, and *Cathelicidin*) and antioxidant (*SOD1, CAT, GPX1, GST, ATOX1, Nrf2* and *AhpC/TSA*) genes expression were significantly up-regulated in supplemented ewes post-lambing.

The periparturient duration of supplementation and the kind of gene had a substantial impact on the mRNA levels of immunological and antioxidant indicators. The higher mRNA levels in the control group were found for *GPX1* (1.09 ± 0.1) and *S-LZ* (1.03 ± 0.2), respectively, pre- and post-lambing. *TLR7* (0.37 ± 0.1) and *AhpC/TSA* (0.37 ± 0.2) had the lowest mRNA transcript levels, in contrast. Regarding the supplemented groups, the higher gene expression trend was detected for *S-LZ* in both pre and post-lambing (1.04 ± 0.3 and 2.1 ± 0.2), while the most decreased level was noticed for *ATOX1* (0.37 ± 0.08) and *NFKB* (1.59 ± 0.3).

**Fig. 3:**
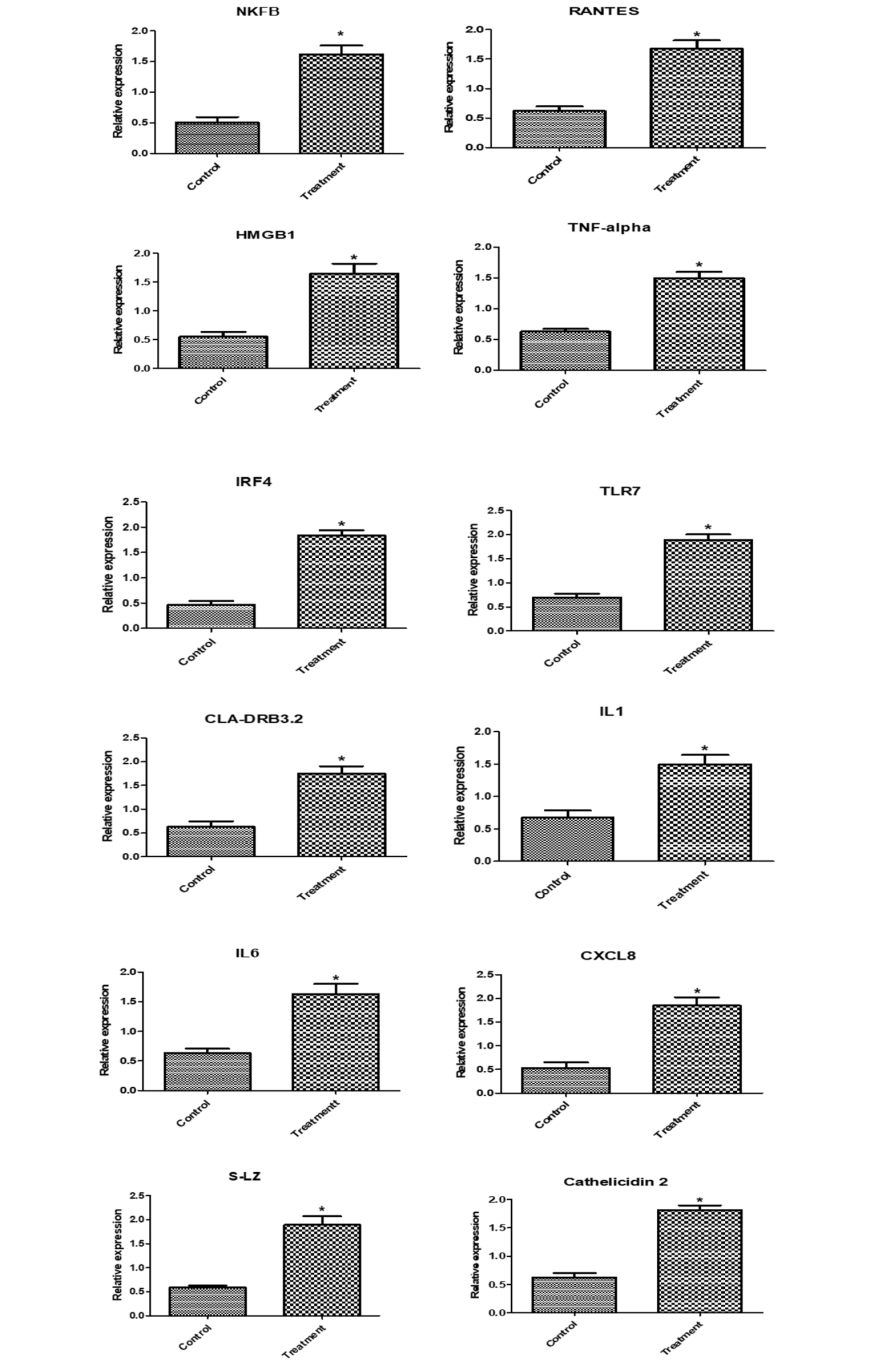
Relative expression patterns of immunity genes in the newly born lambs from Barki ewes supplemented with Nannochloropsis algae. Results are expressed as means ± SEM. *P < 0.05

**Fig. 4:**
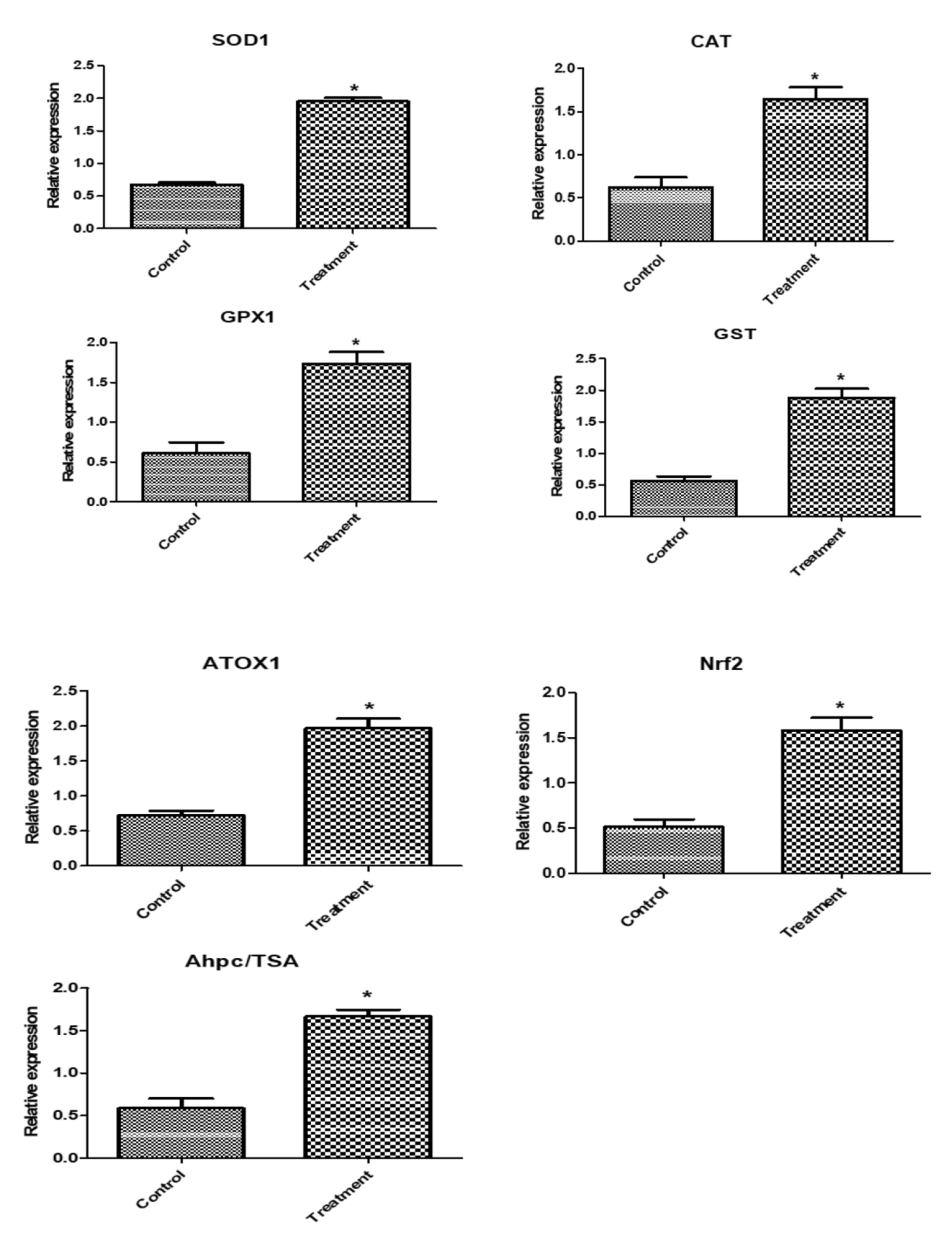
Relative expression patterns of antioxidant genes in the newly born lambs from Barki ewes supplemented with *Nannochloropsis* algae. Results are expressed as means ± SEM. *P < 0.05

**Fig. 5:**
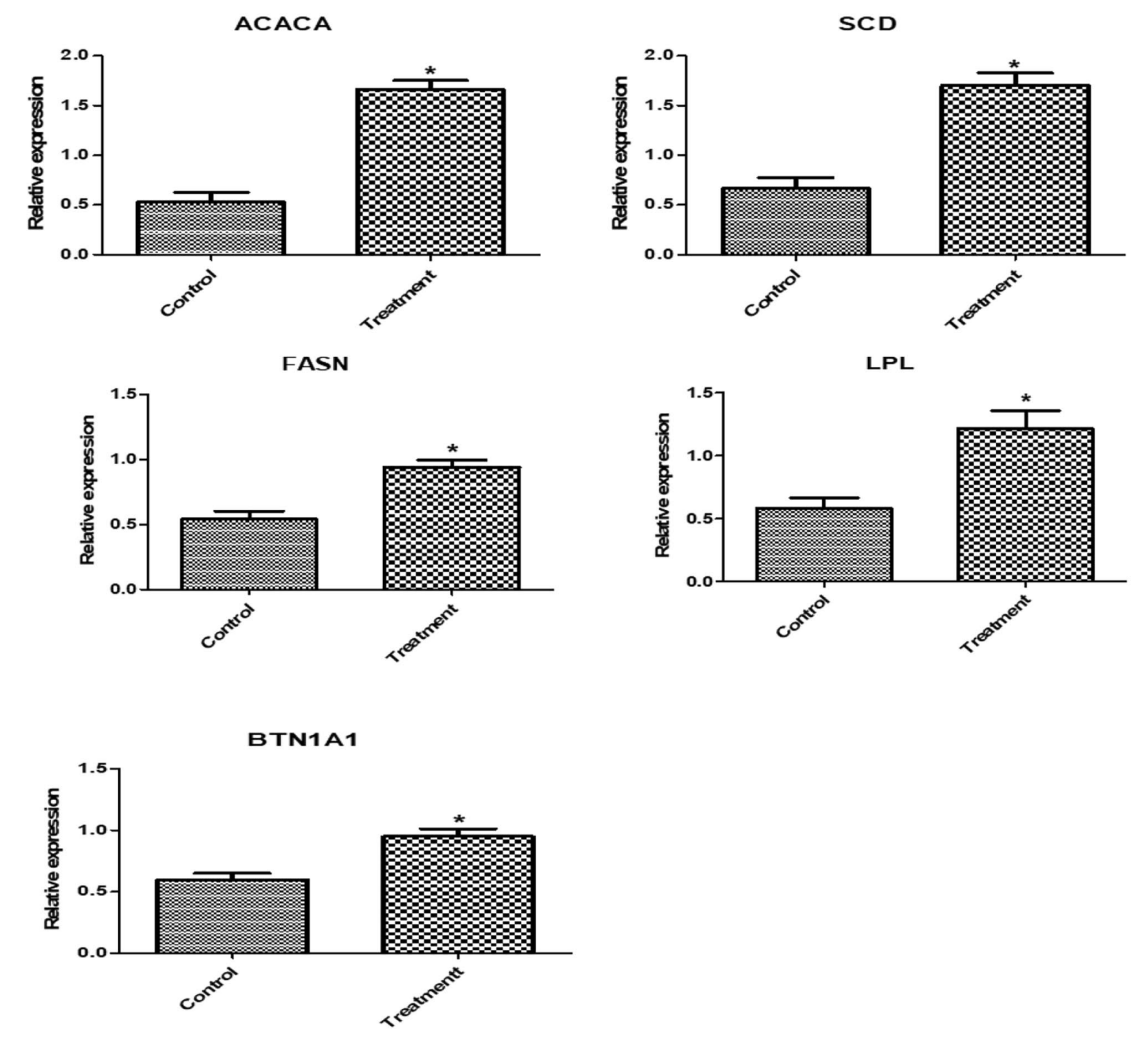
Relative expression patterns of lipogenic genes in the newly born lambs from Barki ewes supplemented with *Nannochloropsis* algae. Results are expressed as means ± SEM. *P < 0.05

Regarding the mRNA levels of investigated markers in newly born lambs, levels of immune (*NFKB, RANTES, HMGB1, TNF-α, IRF4, TLR7, CLA-DRB3.2, IL1B, IL6, CXCL8, S-LZ*, and *Cathelicidin*), antioxidant (*SOD1, CAT, GPX1, GST, ATOX1, Nrf2* and *AhpC/TSA*) and lipogenic (*ACACA, FASN, SCD, LPL*, and *BTN1A*) genes expression were significantly enhanced in newly born lambs from supplemented ewes than control ones (Figs. 3 and 4, and 5). A significant relationship was seen between the gene type and the group to which the lamb was assigned. *ATOX1* had the highest expression profile among the genes examined in the control and supplemented ewe lambs (0.72 ± 0.1 and 1.97 ± 0.2, respectively). The lowest transcript levels were for *FASN* (0.93 ± 0.09) and NFKB (0.5 ± 0.1) genes.

### Effect of Nannochloropsis supplementation on biochemical profile

An overview of hematological, serum biochemical, immunological and antioxidant profile in Barki ewes before and after lambing, after supplementation with *Nannochloropsis*, was illustrated in Table 4. There was a significant (*p* < 0.05) increase in the WBCs, Hb, RBc count (10.3 ± 1.1, 14 ± 0.2 and 12.1 ± 0.4, respectively) in supplemented ewes after lambing as compared with control ones (7 ± 0.05, 10 ± 0.4 and 9.9 ± 0.2), respectively (Table 4). The serum activity of ALT and AST were significantly reduced (*P* = 0.001) with statistically significant high values of creatinine (*P* = 0.027) in control ewes after lambing while the supplemented group with *Nannochloropsis* normalized serum levels of ALT, AST and creatinine. Furthermore, the supplemented group considerably (*P* < 0.05) raised the levels of serum glucose, total protein, GPx, catalae, IL1 α, and IL6 in ewes after lambing, while dramatically lowering the levels of serum TNF-α and MDA in comparison to the equivalent control group. Furthermore, after lambing, nannochloropsis considerably raised the serum levels of total cholesterol and triacylglycerol (*P* < 0.05) in ewes as compared to the control group. Serum levels of albumin, globulin, and urea did not significantly differ across treatments.

**Table 4:** Impact of *Nannochloropsis* algae supplementation on ewes’ metabolic profile before and after lambing. (Mean ± SE).

Parameters	Pre lambing		Post lambing	
	Control	Treatment	Control	Treatment
WBCs (x109/L)	7.7 ± 0.3	7.3 ± 0.1	7 ± 0.05	10.3 ± 1.1*
RBCs(× 1012/L)	10.5 ± 0.5	9 ± 0.2	9.9 ± 0.2	12.1 ± 0.4*
Hb (g/dl)	10.7 ± 0.2	12.1 ± 1	10 ± 0.4	14.6 ± 0.2*
Glucose (mg/dl)	48 ± 1.5	47.6 ± 1.7	45.3 ± 1.4	59.8 ± 2.8*
Cholesterol (mg/dl)	49.6 ± 0.8	33 ± 3.2	51.3 ± 2.3	45.3 ± 2.6*
Triglycerides (mg/dl)	58.6 ± 4.1	67 ± 0.5	52.3 ± 2.1	74.6 ± 2.3*
Total protein (g/dl)	6.1 ± 0.2	5.8 ± 0.08	5.4 ± 0.2	6.6 ± 0.08*
Albumen (g/dl)	1.7 ± 0.3	2.1 ± 0.4	2.1 ± 0. 5	2.4 ± 0.3
Globulin (g/dl)	4 ± 0.2	4.2 ± 0.5	3.7 ± 0. 3	4.2 ± 0.1
Urea (mg/dl)	54.3 ± 1.2	51 ± 3.7	57 ± 4.7	48 ± 0.5
Creatinine (mg/dL)	1.5 ± 0.05	1.5 ± 0.1	2.6 ± 0.1*	1.4 ± 0.2
AST (U/L)	91.3 ± 3.4	84.6 ± 8.4	75.3 ± 0.8*	75.3 ± 2.9
ALT (U/L)	64.3 ± 0.8	47.3 ± 4	47.6 ± 5.7*	46.6 ± 1.7
GPx (U/mL)	1.1 ± 0.02	1.1 ± 0.1	1.2 ± 0.04	2 ± 0.08*
MDA (U/ml)	12.7 ± 2.1	13.4 ± 0.8	9.4 ± 0.3	7 ± 0.2*
Catalase (U/L)	40.3 ± 0.4	47.3 ± 2.8	36.6 ± 2.8	65 ± 0.2*
IL 1α (pg/ml)	6.3 ± 0.8	5 ± 0.4	6.1 ± 0.7	7.4 ± 0.6*
IL 6 (pg/ml)	8.9 ± 0.4	4.6 ± 0.3	8.4 ± 0.3	6.5 ± 0.4*
TNF-α (pg/ml)	31.3 ± 2	33 ± 1.7	30.3 ± 2	22.6 ± 1.2*

*Values with an asterisk within the same raw are statistically significant (*p* < 0.05). WBC: White blood cells; RBC: Red blood cells; Hb: Hemoglobin; AST: aspartate aminotransferase; ALT: alanine transaminase; GPx: Glutathione peroxidase; MDA: Malondialdhyde; IL1 α: interlukin 1 alpha; IL 6: interlukin 6; TNF-α: tumer necrosis factor alpha

Table 5 displays the impact of supplementing late-pregnant Barki ewes with *Nannochloropsis* on the performance, metabolic profile, and survivorship of their offspring. When late pregnant ewes supplemented with *Nannochloropsis*, their lambs’ blood levels of TNF-α and stillbirth (47.6 ± 1.7 pg/ml and 10%) were significantly (*P* < 0.05) lower than those in the control groups (68.3 ± 3.2 pg/ml and 30%) respectively, while their WBCs, Hb, RBc count, birth weight, and body temperature were significantly (*P* < 0.05) higher.

**Table 5:** Impact of *Nannochloropsis* algae supplementation on newly born lambs’ survivability, performance and metabolic profile (Mean ± SE).

Parameters	Lambs	
	Control	treatment
Lamb birth weight	2.9 ± 0.05	3.3 ± 0.03*
Lamb temperature	37.9 ± 0.05	38.6 ± 0.06*
Stillbirth (%)	30% (3/10)	10% (1/10) *
WBCs (x109/L)	6.9 ± 0.5	10.1 ± 0.05*
RBCs(× 1012/L)	9 ± 0.17	11.9 ± 0.4*
Hb (g/dl)	8 ± 0.2	14.1 ± 0.1*
Total protein (g/dl)	5 ± 0.2	5.2 ± 0.5
Albumen (g/dl)	2 ± 0.2	2.2 ± 0.2
Globulin (g/dl)	3 ± 0.05	2.8 ± 0.8
AST (U/L)	55 ± 6.4	45.6 ± 3.3
ALT (U/L)	35.6 ± 2.3	30.3 ± 1.4
TNF-α (pg/ml)	68.3 ± 3.2	47.6 ± 1.7*

*Values with an asterisk within the same raw are statistically significant (*p* < 0.05). WBC: White blood cells; RBC: Red blood cells; Hb: Hemoglobin; AST: aspartate aminotransferase; ALT: alanine transaminase; TNF-α: tumer necrosis factor alpha

### Correlation between gene expression pattern and serum profile of biochemical markers in supplemented Barki ewes

#### Pre-lambing

There was a negative correlation found between the serum concentrations of catalase and the mRNA levels of *SOD1* (*r*= -0.999 and *p* = 0.027), a certain correlation between the serum values of GPX and the mRNA levels of *TNFα* (*r* = 1 and *p* = 0.015), an antagonist correlation between the serum concentrations of TNFα and the mRNA levels of *IRF4* (*r*= -1 and *p* = 0.004), and a firm correlation between the serum levels of cholesterol and the mRNA levels of *HMG1* and *AhpC/TSA* (*r* = 0.999 and *p* = 0.02, *r* = 0.998 and *p* = 0.035, respectively).

Serum concentrations of creatinine were differntly correlated with mRNA levels of *NFKB1*, serum values of triglyceride were contrairely correlated with mRNA levels of *CLA-DRB3.2* and *GST* (*r*= -0.998 and *p* = 0.035, *r*= -0.998 and *p* = 0.04, respectively), serum concentrations of albumen were undoubtedly correlated with mRNA levels of *HMG1*, serum levels of urea were emphatically correlated with mRNA levels of *cathelicidin* (*r* = 1 and *p* = 0.008) and negatively correlated with mRNA levels of *CAT* (*r*= -1 and *p* = 0.002), serum levels of globulin were adversely correlated with mRNA levels of *TNFα* (*r*= -0.999 and *p* = 0.03), serum levels of total protein were positively correlated with mRNA levels of *CLA-DRB3.2* and *GST* (*r* = 1 and *p* = 0.003, *r* = 1 and *p* = 0001, respectively) and adversly correlated with mRNA levels of *Nrf2* (*r*= -0.997 and *p* = 0.05).

#### Post-lambing

The serum concentrations of catalase were positively correlated with mRNA levels of *TLR7* and *CAT* (*r* = 0.999 and *p* = 0.04, *r* = 1 and *p* = 0.007, respectively) and adversly correlated with mRNA levels of *SOD1* (*r*= -0.998 and *p* = 0.03), serum values of GPX were differently correlated with mRNA levels of *GPX1* and *Nrf2* (*r*= -0.998 and *p* = 0.048, r- -0.998 and *p* = 0.04, respectively), serum levels of MDA were certainly correlated with mRNA levels of *RANTES* and *GST* (*r* = 1 and *p* = 0.004, *r* = 0.999 and *p* = 0.03, respectively).

Serum values of IL6 were undoubtedly correlated with mRNA levels of *RANTES*, *CXCL8* and *GST* (*r* = 1 and *p* = 0.008, *r* = 0.999 and *p* = 0.02, *r* = 0.999 and *p* = 0.02, respectively), serum concentrations of glucose were emphatically correlated with mRNA levels of *TLR7* and *CAT* (*r* = 1 and *p* = 0.004, *r* = 0.999 and *p* = 0,02 respectively) and differntly correlated with mRNA levels of *cathelicidin* (*r*= -0.997 and *p* = 0.04), serum concentrations of cholesterol were firmly correlated with mRNA levels of *cathelicidin*, *SOD1* and *CAT* (*r* = 0.999 and *p* = 0.02, *r* = 1 and *p* = 0.002, *r* = 0.997 and *p* = 0.04, respectively), serum levels of urea were certainly correlated with mRNA levels of *IL1β* ((*r* = 0.999 and *p* = 0.02), serum levels of AST were positively correlated with mRNA levels of *RANTES*, *CXCL8* and *GST* (*r* = 0.999 and *p* = 0.02, *r* = 1 and *p* = 0.007, *r* = 1 and *p* = 0.0001, respectively). Serum levels of ALT were contrairely correlated with mRNA levels of *NKFB, CXCL8, GST* and *ATOX1* (*r*= -0.998 and *p* = 0.03, *r*= -0.998 and *p* = 0.04, *r*= -0.997 and *p* = 0.04, *r*= -0.999 and *p*= -0.03, respectively), serum values of globulin were positively correlated with mRNA levels of *NFKB* and *ATOX1* (*r* = 1 and *p* = 0.02, *r* = 0.999 and *p* = 0.02, respectively).

### Correlation between gene expression pattern and serum profile of biochemical markers in newly born lambs from supplemented Barki ewes

The serum values of TNFα were positively correlated with mRNA levels of *RANTES* (*r* = 0.999 and *p* = 0.02) and contrairly correlated with mRNA levels of *cathelicidin* (*r*= -1 and *p* = 0.01), serum concentrations of albumen were negatively correlated with mRNA levels of *NFKB* (*r*= -1 and *p* = 0.01), serum levels of total protein were firmly correlated with mRNA levels of *SL-Z* (*r* = 1 and *p* = 0.01), serum values of AST were certainly correlated with mRNA levels of *HMG1* (*r* = 0.999 and *p* = 0.02) and differntly correlated with mRNA levels of *IL6* and *SOD1* (*r*= -0.997 and *p* = 0.04, *r*= -0.999 and *p* = 0.02), serum concentrations of ALT were undoubtedly correlated with mRNA levels of *IL1β* (*r* = 0.998 and *p* = 0.03) and serum levels of globulin were positively correlated with mRNA levels of *SL-Z* and *ATOX1* (*r* = 0.999 and *p* = 0.02, *r* = 0.997 and *p* = 0.04, respectively).

## Discussion

As far as we know, there have been very few research done on the possible impact of supplementing with *Nannochloropsis* on the gene expression and serum profile of immune, antioxidant, and lipogenic markers in transitional Barki ewes. The current investigation showed that taking supplements containing *Nannochloropsis* stimulates antioxidant activity, enhances immunity, and produces favorable energetic properties.

Our findings revealed that supplementation of ewes with *Nannochloropsis* significantly agumented the expression pattern of immune and antioxidant markers in ewes post-lambing as well as their newly born lambs. Additionally, mRNA levels of lipogenic markers were significantly increased in lambs from supplemented ewes than control ones. Our study is the first to explore the alterations in gene expression profile of immune and antioxidant markers as a result of supplementation with *Nannochloropsis* microalgae in sheep (*Ovis aries*).

Previous research examined lipogenic genes expression profiles to track the health state of sheep after supplementing with various algae. For instance Fan et al. 2019 cited that algae supplementation altered the expression of lipid metabolism related genes in sheep managed under intensive finishing system. In addition, the effects of addition of marine algae to the diet on adipose tissue development, fatty acid profile, lipogenic gene expression, and meat quality in lambs reported by (Urrutia et al. 2016). In other livestock, the effect of microlagae on the transcript levels of immune genes was reported; where the oral supplementation with cyanobacterium *Spirulina platensis* and *Chlorella vulgaris* up-regulated *IL-8* and *IL-1β* respectively in the ileum of piglets around weaning without changes in *TNF-α, IL-1β, IL-10* and *TGF-β* elucidated by (Furbeyre et al. 2018). In seabream, orally administration of *Tetraselmis chuii* induced an increase in expression levels of several genes associated to immune system, such as T-cell receptor beta (*TCR-β*), major histocompatibility complex genes and IgM (Cerezuela et al. 2012).

Based on our findings, supplementing ewes and their lambs with *Nannochloropsis* enhanced the transcript levels of immunological markers that have been investigated (*NFKB, RANTES, HMGB1, TNF-α, IRF4, TLR7, CLA-DRB3.2, IL1B, IL6, CXCL8, S-LZ*, and *Cathelicidin*). In inflammatory circumstances, serum cytokines like (IL1B, IL6, TNF-α, and NFKB) serve as indirect markers (Salim et al. 2016). One of the most important pro-inflammatory cytokines in the immune response is TNF-α. B lymphocytes, T lymphocytes, NK (natural killer) lymphocytes, and LAK (lymphokine-activated killer) cells are just a few of the immune system cells that TNF-α activates, along with other substances (Benedict et al. 2003). TNF-α also triggers the release of a wide variety of other cytokines (Bradley 2008). On chromosome BTA23q22, the gene that codes for *TNF-α* has four exons and three introns (Lester et al. 1996). Many different kinds of mammalian cells express it, although macrophages and monocytes do so most potently. Lipopolysaccharide (LPS), which is present in the bacterial cell wall, stimulates the production of TNF-α in these phagocytic cells. According to (Bannerman 2009), *TNF-α* gene expression triples in LPS-stimulated macrophages, mRNA levels rise by about 100-fold, and the protein itself may be secreted at a rate of up to 10,000 times greater. According to research by (Fremond et al. 2004), NFKB activation and cytokine production aid in bacterial identification.

Many cells, including blood lymphocytes, express the chemokine regulated on activation normal T-cell expressed and secreted (RANTES), also known as CCL5 chemokine, in response to inflammatory signals (Oliva et al. 1998). According to (Taub et al. 1995), it controls the activation and movement of both inflammatory and non-inflammatory cells. It also has a role in the acute phase response (Tavares and Miñano 2004). The expression of interferon-inducible genes that play roles in the immune response is regulated by the interferon regulatory factor (IRF) family of DNA-binding proteins. According to (Do et al. 2010), it also affects how B and T cells differentiate.

Leucine-rich repeat domain (LRR) domains, which are crucial structures that identify pattern-recognition receptors (PAMPs) from other molecules, are reported to make up the majority of the ectodomains of TLR molecules (Botos et al. 2011). Regions encoding LRR domains are notably abundant in non-synonymous SNPs, according to comparisons of SNP distribution in TLR coding regions in several mammals (White et al. 2003). Non-synonymous SNPs in the LRR domains have the potential to significantly change a molecule’s capacity to recognize external pathogens (Fujita et al. 2003). According to (Karrow et al. 2014), the *MHC* genes are significant possibilities for disease resistance. The caprine *MHC* gene, commonly referred to as caprine lymphocyte antigen (CLA) or goat lymphocyte antigen (GoLA), is found on chromosome.23 MHC class I, MHC class II, and MHC class III are its three subgroups. By delivering external antigens to helper T-lymphocytes, the class II molecule among these is crucial in the beginning of the immune response (Li et al. 2006). It is further divided into the DQ and DR subtypes (Takada et al. 1998). The DRB locus is the more polymorphic of these two kinds (Schook and Lamont 1996) and is functionally in charge of individual variations in the immune response to infectious pathogens (Dukkipati et al. 2006).

Leucocytes have a variety of antimicrobial defense, including cathelicidins. Seven bovine cathelicidin genes with proven expression of peptides possessing the antibacterial activity have been identified, as summarized by (Bagnicka et al. 2010) and several of them were found in milk from mastitic mammary glands (Strzałkowska and Jo 2012). High-mobility group box 1 (HMGB1) is a highly abundant and conserved protein with significant biological functions. It can activate the toll-like receptor 4 (TLR4) to produce cytokines or stimulate the CXCR4 to enhance chemotaxis by binding to the chemokine CXCL12 (Magna and Pisetsky 2014). Pathogen-associated molecular patterns (PAMPs), cytokines, and chemokines interact with HMGB1 to extend its extracellular effects beyond its intrinsic activity (Andersson and Harris 2010).

The C-X-C motif chemokine ligand 8 (*CXCL8*) gene, also known as interleukin 8 (IL8), is a member of the chemokine family. It is well known for its ability to chemotactically attract leukocytes and lymphocytes, and it is essential for inflammation, immune responses, and organism defence (Heinzmann et al. 2004). Endothelial cells, macrophages, and monocytes are the main producers of CXCL8. By encouraging neutrophil activation, migration, adhesion, and phagocytosis from the peripheral circulation to the tissues, CXCL8’s primary function is to start and intensify the inflammatory response brought on by pathogens (Velloso et al. 2005). According to (Jundi and Greene 2015), CXCL8 also possesses chemotactic activity against basophils and T cells. According to the our findings and the previous interpretations, we could postulate that adding *Nannochloropsis* had a positive impact on immune function of sheep via enhancing the expression profile of immune genes.

In the same line, our results showed that adding *Nannochloropsis* to ewes and their lambs improved the transcript levels of antioxidant (*SOD1, CAT, GPX1, GST, ATOX1, Nrf2* and *AhpC/TSA*) markers. Antioxidants fight free radicals by scavenging them, detoxifying them, preventing their generation, or sequestering the transition metals that produce (Masella et al. 2005). These mechanisms include endogenous antioxidant defences made by the body, such as SOD, CAT, and glutathione peroxidase (GPx), as well as non-enzymatic antioxidant defences (Glasauer and Chandel 2014). Hydrogen peroxide (H2O2) can be catalysed by the peroxiredoxin (PRDX) family of antioxidant enzyme oxido-reductase proteins thanks to a conserved ionised thiol.

The *ATOX1* gene produces the copper metallochaperone protein known as ATOX1 (Klomp et al. 1997). ATOX1 guarded against reactive oxygen species in cells. Due to the fact that it transfers copper from the cytosol to the transporters ATP7A and ATP7B, ATOX1 is essential for maintaining copper homeostasis (Maret and Wedd 2014). Thiol-specific peroxidase functions as a sensor of hydrogen peroxide-mediated signalling events and contributes to cellular defence against oxidative stress by detoxifying peroxides and sulfate-containing radicals. The main inducible defence against oxidative stress is the Nrf2 stress response system, which controls the production of cytoprotective genes (Yamamoto et al. 2018). We may hypothesize that the addition of *Nannochloropsis* improved the expression profile of antioxidant genes, hence improving the antioxidant function of sheep, based on our data and earlier interpretations.

The mechanism of increasing the expression profile of immune and antioxidant in Barki ewes supplemented with *Nannochloropsis* microalgae during peri-parturient period and their newly born lambs could be explained by the polysaccharides from microalgae have been shown to activate immune responses from immune cells by binding to toll-like receptors (Balachandran et al. 2006). Previous research has shown that supplementing small ruminants with microalgae increases their antioxidant activity (EL-Sabagh et al. 2014). Furthermore, it was claimed that microalgae had stronger immunological and antioxidant qualities since they include phenolic substances such beta-carotene zeaxanthin, α--tocopherol, and phycocyanins (Bhatt et al. 2012). Additionally, it was proposed by that spirulina boosts the immune response, particularly the initial response, by promoting the activities of macrophages, phagocytosis, and IL-1 production.

Regarding the mRNA levels of lipogenic markers, it was noticed that the lipogenic (*ACACA, FASN, SCD, LPL*, and *BTN1A*) genes were significantly enhanced in newly born lambs from *Nannochloropsis* supplemented ewes. Key lipogenic enzymes such as acetyl-CoA carboxylase alpha (ACACA), fatty acid synthase (FASN), stearoyl-CoA desaturase (SCD), lipoprotein lipase (LPL), and butyrophilin subfamily 1 member A1 (BTN1A1) have been the focus of research to determine the factors that control their activity in ruminant species (Bernard et al. 2009). ACACA, FASN and SCD have been studied in Barki ewes, lactating sheep (Ticiani et al. 2016) and in different sheep breeds (Izadi et al. 2016). According to (Van Harten et al. 2013), the variation in gene expression of a number of regulatory enzymes of the intermediate metabolism can offer helpful tools to enhance genetic selection for cattle adaptability to challenging conditions. It was proved that adding algae to the diet was easier to obtain docosahexaenoic (DHA) deposition than through the desaturation and prolongation pathway of α-Linolenic acid (Urrutia et al. 2016); therefore it could decipher the modulation in the expression profile of lipogenic genes. According to the previously mentioned results adding *Nannochloropsis* could promote the growth of the young lambs.

The method of augmenting the lipogenic genes expression profile in lambs derived from Barki sheep fed *Nannochloropsis* microalgae may be clarified by the fact that microalgae are rich in minerals, carotenoids, fatty acids, especially gamma-linolenic acid, which has many health benefits, and all important amino acids, including vitamin A (Howe et al. 2006). Additionally, it has been demonstrated that microalgae can alter the composition of the bacterial population and reduce the breakdown of rumen proteins, both of which boost the effectivly of rumen microbial crude protein production (Panjaitan et al. 2010). Along the same lines, the impacts of microalgae on ruminal (volatile fatty acids) VFAs altered rumen fermentation increased the amount of energy available for development, and thus boosted production (Boeckaert et al. 2007). Additionally, microalgae may enhance animal growth by increasing feed consumption, feed conversion, nutrient uptake and utilization, and body weight gain (Evans et al. 2015). According to Bernabucci et al. 2005; there may be a connection between body weight and oxidative status, suggesting that microalgae’s antioxidant qualities contributed to the pronounced up-regulation of lipogenic markers.

upplementation of late pregnant ewes with *Nannochloropsis* induced a significant increased in WBCs, Hb, RBc count in the supplemented ewes and their newly born lambs as compared with the control group. The present study’s findings were in line with those of (EL-Sabagh et al. 2014) for fattening lambs and (Ghattas et al. 2019) in calves, but they differed from those of (Alazab et al. 2020) who discovered that all tested hematological parameters of growing rabbits treated with Spirulina platensis showed non-significant improvements when compared to the control group. Leukocytes play a vital role in nonspecific or innate immunity, and their numbers can be viewed as indicators of relatively low susceptibility to disease (Matanović et al. 2007). The high concentration of polysaccharide components, folic acid, and vitamin B12, as well as *Nannochloropsis’s* superior absorption of these nutrients, may account for the plant’s positive effects on hematological parameters (Nedeva et al. 2014).

There was a significant increase in glucose level in supplemented ewes with *Nannochloropsis* as compared with control ones which could be attributed to *Nannochloropsis* is rich in vitamin A, which increase intestinal glucose absorption and enhance insulin release and sensitivity (Rhee and Plutzky 2012). These results were in contrary with that found in rabbits (Abd El-Hamid et al. 2022) and in goats (Kholif et al. 2020) who found non-significant differences in serum glucose concentrations among groups. Therefore, supplementing late pregnant ewes with Nannochloropsis improved their energy status; reduced stress in ewes caused by lamb delivery, and had beneficial effects on their health postpartum (ABD ELDAIM et al. 2018).

Our results showed significant increase of total protein in supplemented ewes compared to control ones. These outcomes are in line with other studies on dairy Zaraibi goats (Khalifa et al. 2016), but unlike to the finding reported by (ABD ELDAIM et al. 2018) who found non-significant improvement of total protein level of ewes treated with Spirulina platensis compared to the control group. The high levels of protein, essential amino acids, vitamins, minerals, phospholipids, and antioxidants found in *Nannochloropsis* were linked to a substantial increase in total protein in supplemented ewes compared to control ewes in the current study (Schulze et al. 2016).

There was a meaningful elevation in serum level of cholesterol and triglycerides in supplemented ewes with *Nannochloropsis* as compared with control ewes. These results were inconsistency with previous reports in ewes (ABD ELDAIM et al. 2018). However, in contrast to what was noted for Zaraibi goats (Khalifa et al. 2016), cholesterol levels in Zaraibi goats supplemented with *Spirulina platensis* were significantly reduced compared to the control group. The findings in mice given a high-cholesterol diet demonstrate a negative relationship between blood cholesterol levels and the amount of cysteine present in dietary proteins. The authors attribute this relationship to *spirulina’s* capacity to elevate cholesterol levels and its high cysteine content in the phycocyanin protein (Vedi et al. 2013). The considerable increase in serum levels of triglycerides and cholesterol in supplemented sheep compared to control ones was caused by the high lipid content of *Nannochloropsis* (De Morais et al. 2015).

Reduced activity of serum ALT and AST, increased levels of creatinine and TNF-α, and consistently lower glucose concentrations in control ewes during lambing could point to malnourishment or metabolic stress related to lactation and lambing. Malnutrition may be a risk factor for several metabolic and hepatic illnesses linked to decreased ALT and AST activity. Elevated creatinine levels in late-pregnant sheep are linked to subclinical/clinical ketosis (Van Saun 2000) and the inflammatory cytokine TNF-α (El-Deeb 2012). These outcomes were in line with earlier goat, (Yadav, Kumar) calves (Ghattas et al. 2019), fattening lambs (ABD ELDAIM et al. 2018; EL-Sabagh et al. 2014), cow (Wullepit et al. 2012), rabbits (Abd El-Hamid et al. 2022) and in broilers (Abdel-Moneim et al. 2022; Elbaz et al. 2022). The treatment of *Nannochloropsis* to unsupplemented Baki ewes lowered their risk of metabolic problems and oxidative stress, as indicated by decreased glucose concentrations and raised TNF-α levels. *Nannochloropsis* supplementation resulted in normalized creatinine, ALT, and AST levels in pregnant Barki ewes, as well as decreased TNF-α and MDA levels and raised CAT and GPx levels.

GPx represents the first line of defense in cellular antioxidant mechanisms (Ighodaro and Akinloye 2018), level of MDA indicates the extent of lipid peroxidation in living cells (Mesalam et al. 2021), and the process of extracting these peroxides and turning them into O2 involves CAT in the second stage (Yu 1994). The fact that mitochondria are intricate organelles with the ability to produce intracellular reactive oxygen species is widely known (Vakifahmetoglu-Norberg et al. 2017). Increased ROS levels can lead to oxidative stress when mitochondrial ROS production exceeds the cellular antioxidant capacity (Liemburg-Apers et al. 2015). The potential of *Nannochloropsis* to alleviate oxidative stress by lowering oxidative markers like MDA and carbonyl protein (Bendaoud et al. 2019) and its antioxidant-rich content (carotenoids, fucoxanthin, astaxanthin, and vitamins) to scavenge free radicals and prevent lipid peroxidation (Yaakob et al. 2014) may also account for the enhanced antioxidant defense mechanisms in *Nannochloropsis*-supplemented ewes.

The supplemented ewes exhibited a significant rise in IL1α and IL-6 levels as compared to the control group. Our finding was similar to that obtained by (Ghattas et al. 2019). It is possible that these changes are caused by beneficial molecules found in the microalga that have been demonstrated to have immune-stimulatory activity, such as polysaccharides rich in (β1→3, β1→4)-glucans, (α1→3)-, (α1→4)-mannans, and anionic sulphated heterorhamnans, as well as sulfated lipids, polyunsaturated fatty acids, and astaxanthin. These compounds can all stimulate dendritic cells (Manzo et al. 2019), T-cells (Chuang et al. 2014), or macrophage cells (Chen et al. 2019). These compounds exhibit great promise as molecular adjuvants (Manzo et al. 2019) and can stimulate immune responses and maturation (Carolina et al. 2019).

The current study found that supplementing late-pregnant Barki ewes with nannochloropsis at the late stage of pregnancy raised the body temperature and birth weight of the lambs, while lowering the percentage of stillbirths and blood levels of TNF-α. These results were associated with the high nutritional density of *Nannochloropsis* and the extracellular enzyme release promoted by the gut microbiota (Tovar et al. 2002). Furthermore, *Nannochloropsis* has a variety of nutrients, such as vitamins, minerals, amino acids, essential fatty acids, and other elements that could hasten growth (Khan 2018).

There was a significant rise in rectal temperature of the newborn lambs from the supplemented group as compared to control ones. These results were consistent with the work of ABD ELDAIM et al. (2018). They found that newborn lambs in the *spirulina platensis* algae supplemented pregnant ewes had higher rectal temperatures than the control group. Our findings could be explained by *Nannochloropsis’s* high vitamin A content, which has been demonstrated to boost the expression of uncoupling protein, a brown adipose tissue mitochondrial protein. By converting the energy from nutrition metabolism into heat (a process known as nonshivering thermogenesis), this protein aids in warming the newborn animal (Bonet et al. 2000). Consequently, supplementing late-pregnant Barki ewes with nannochloropsis helps shield the newborn lambs from hypothermia, which is a major cause of neonatal animal death because it inhibits nursing and reduces brown fat stores (Rook et al. 1990).

Ultimately, the addition of *Nannochloropsis* to the late pregnant Barki ewes decreased the mortality rate of their newborn lambs (stillbirth) by 10% in comparison to the newborn lambs from the control ewes (30%). The presence of advantageous molecules in the microalga, such as proteins, S-nucleotide adenosyl peptide complex, polysaccharides, and phenolic compounds, is the reason behind the decline in the mortality rates of newborn lambs (Abd El-Hamid et al. 2022). In addition to its pigment content of violaxanthin with β-carotene and vaucheriaxanthin, it has excellent nutritional value, antimicrobial, anti-inflammatory, antioxidant, and immune-stimulatory properties (Md et al. 2018). Apart from the ability of microalga to increase the concentrations of probiotics in the gastrointestinal tracts (Mahmood et al. 2017). Furthermore, microalgae’s ability to reduce the pathogenic bacteria in animals (Abedin and Taha 2008).

Additionally, *N oculata* is thought to be a promising alga for aquaculture because of its ability to accumulate high concentrations of polyunsaturated fatty acids, which have been demonstrated to improve the vigor of newborn lambs, possibly by lengthening the gestation period or promoting better development of the foetus’s neural tissue (Toral et al. 2010).

## Conclusion

When given *Nannochloropsis* supplements, Barki ewes and their newborn lambs have improved immunological, antioxidant, and metabolic profiles. Immune and marker expression patterns were shown to be significantly up-regulated in post-lambing ewes and their newborn lambs. Lambs from supplemented ewes also had considerably higher mRNA levels of lipogenic markers. There was also evidence of immune-modulatory and antioxidant improvement of the immunological and antioxidant biochemical profile. An efficient method for improving the immune and antioxidant status of late-pregnant Barki ewes and their lambs could be thought of as prepartum supplementation with *Nannochloropsis*.

## Data Availability

No datasets were generated or analysed during the current study.

## Abbreviations

WBC: White blood cells
RBC: Red blood cells
Hb: Hemoglobin
AST: Aspartate aminotransferase
ALT: Alanine transaminase
GPx: Glutathione peroxidase
MDA: Malondialdhyde
IL1 α: Interlukin 1 alpha
IL 6: Interlukin 6
TNF-α: Tumer necrosis factor alpha
